# Structural models of the NaPi-II sodium-phosphate cotransporters

**DOI:** 10.1007/s00424-018-2197-x

**Published:** 2018-09-03

**Authors:** Cristina Fenollar-Ferrer, Lucy R. Forrest

**Affiliations:** 1 Laboratory of Molecular and Cellular Neurobiology, National Institutes of Mental Health, National Institutes of Health, Bethesda, MD 20892 USA; 2 Laboratory of Molecular Genetics, National Institute on Deafness and Other Communication Disorders, National Institutes of Health, Bethesda, MD 20892 USA; 3 Molecular Biology and Genetics Section, National Institute on Deafness and Other Communication Disorders, National Institutes of Health, Bethesda, MD 20892 USA; 4 Computational Structural Biology Section, National Institutes of Neurological Disorders and Stroke, National Institutes of Health, Bethesda, MD 20892-3761 USA

**Keywords:** Transporter, Structure prediction, Homology modeling, Inverted-topology repeats, Repeat-swap modeling, Hidden Markov models

## Abstract

Progress towards understanding the molecular mechanisms of phosphate homeostasis through sodium-dependent transmembrane uptake has long been stymied by the absence of structural information about the NaPi-II sodium-phosphate transporters. For many other coupled transporters, even those unrelated to NaPi-II, internal repeated elements have been revealed as a key feature that is inherent to their function. Here, we review recent structure prediction studies for NaPi-II transporters. Attempts to identify structural templates for NaPi-II transporters have leveraged the structural repeat perspective to uncover an otherwise obscured relationship with the dicarboxylate-sodium symporters (DASS). This revelation allowed the prediction of three-dimensional structural models of human NaPi-IIa and flounder NaPi-IIb, whose folds were evaluated by comparison with available biochemical data outlining the transmembrane topology and solvent accessibility of various regions of the protein. Using these structural models, binding sites for sodium and phosphate were proposed. The predicted sites were tested and refined based on detailed electrophysiological and biochemical studies and were validated by comparison with subsequently reported structures of transporters belonging to the AbgT family. Comparison with the DASS transporter VcINDY suggested a conformational mechanism involving a large, two-domain structural change, known as an elevator-like mechanism. These structural models provide a foundation for further studies into substrate binding, conformational change, kinetics, and energetics of sodium-phosphate transport. We discuss future opportunities, as well as the challenges that remain.

## Introduction

### Biological roles of NaPi-II transporters

**Fig. 1:**
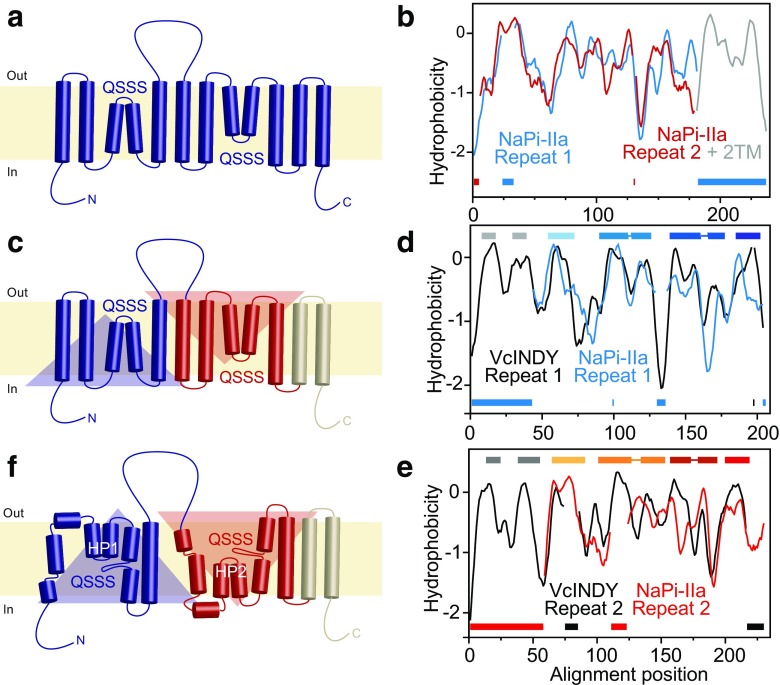
Evolution of the predicted transmembrane topology of the NaPi-II transporters. **a** Biochemical assays identified the cytoplasmic orientation of the N- and C-terminal domains, the extracellular location of the long central loop, and the opposite accessibility of the repeated QSSS motif in NaPi-IIb; together with hydrophobicity analysis, these data indicated a transmembrane topology with a total of 12 membrane-spanning segments. **b** Identification of the extents of the inverted-topology repeats was achieved by alignment of two halves of the family-averaged hydropathy plot, after dividing at the position of the large extracellular loop. The last two transmembrane segments are predicted to be peripheral. **c** Updated transmembrane topology after identification of the repeat elements. **d**, **e** Identification of a relationship between the structural repeats in VcINDY and the sequence repeats in NaPi-IIa, based on alignment of the family-averaged hydropathy profiles. Insertions are indicated below the profiles using the same colors as the profiles themselves. Regions of transmembrane helices are indicated above the profiles as colored bars. **f** Transmembrane topology of a homology model of NaPi-II built using VcINDY as a template, with a total of eight membrane-spanning segments, plus two re-entrant helical hairpins called HP1 and HP2, which originate from, and return to, the extracellular and cytoplasmic sides of the membrane, respectively. The QSSS motifs are predicted to be located in non-helical segments of TM2 and TM5

Phosphate has key metabolic and structural roles in living organisms. Levels of inorganic phosphate (P_i_) are tightly controlled in the cell body to ensure correct function. Misregulation of the pathways that control phosphate homeostasis in the body can lead to severe disorders, such as bone mineralization, soft tissue calcification, or renal lithiasis. The kidney plays a central role in this homeostasis by facilitating the reabsorption of phosphate in the proximal tubule; this reabsorption is mediated by transporters belonging to the solute carrier family SLC34, also known as NaPi-II cotransporters, which use the sodium-electrochemical gradient to drive phosphate translocation against its concentration gradient [5, 15, 25]. The three members of the SLC34 family, NaPi-IIa, b, and c, differ in their Na^+^:P_i_ stoichiometry; members NaPi-IIa and NaPi-IIb are electrogenic and transport three sodium ions per phosphate molecule, while NaPi-IIc is electroneutral, transporting two sodium ions per phosphate [17]. During the last 20 years, a large amount of experimental data including cysteine scanning mutagenesis, epitope labeling, and in vitro glycosylation assays has been amassed (see Forster IC, in this issue). These data were combined with hydropathy profiles, in which the protein sequence was converted to an averaged hydrophobicity to identify likely membrane-spanning segments, leading to the proposal of a common transmembrane topology for NaPi-II transporters [14, 21]. This topology comprised 12 transmembrane helices arranged into two segments of opposing orientations that are flanked by cytoplasmic N- and C-terminal domains (Fig. 1a). The presence of such so-called inverted-topology repeats has been observed in a majority of secondary active transporters for which structures have been determined, and these repeats can underlie the alternating access mechanism that governs transport [10].

Despite the abundance of biochemical and electrophysiological data, no three-dimensional structural data is available for this family of transporters, hindering progress towards a detailed mechanism of transport. In such situations, computational techniques to predict protein structures or to analyze the amino acid sequences of related proteins can prove a valuable stopgap, aiding in the interpretation of the available structure-functional data and leading to new, experimentally testable hypotheses.

### Advances in modeling tools

The accuracy of predicted protein structures depends primarily on the level of information available for structural homologs of the protein of interest, or target. If the structure of a close homolog of the target has been determined to high resolution, then that structure can be used as a template during a procedure known as homology modeling, in which the most similar regions of the protein structure are essentially copied, while more dissimilar regions are adjusted or inserted according to physicochemical or empirical rules of protein structure. Assuming that the appropriate relationship between the template and target proteins has been identified, namely, by accurate alignment of their primary sequences, available methods for homology modeling can construct protein models with high accuracy [22]. For membrane proteins, whose structures diverge less during evolution than those of their water-soluble counterparts due to the constraints imposed by the membrane, the reliability of homology models is particularly high. For example, when the sequence alignment between the template and target proteins contains > 40% identical residues, models built from those alignments are likely to be correct within ~ 1 Å of the native structure, at the level of the protein backbone in the transmembrane segments [11, 29]. As the similarity of the target to proteins of known structure decreases, however, several challenges arise. First, identification of the appropriate template structure becomes more difficult. Second, the likelihood of obtaining a reasonable alignment between the sequences of the target and template decreases. Finally, even in cases where the two proteins clearly share the same overall architecture, i.e., the same number, length, and spacing of transmembrane segments, the probability that the protein adopts a similar structure also diminishes. Thus, for two proteins sharing 10% identical residues, the expected accuracy of the model can be as impressive as 1.5 Å or as low as 3.5 Å, considering only the backbone atoms in the transmembrane helices [11, 29]. And, of course, one has no way of knowing where on this spectrum, the current prediction lies.

The strategy of structure prediction by homology modeling as discussed so far assumes the availability of at least one structure of similar architecture. In the absence of such a template, a number of procedures have been developed that either assemble fragments of known structure or use evolutionary information from sequence homologs to identify constraints that, in turn, are used to guide model-building. Both of these template-free methods typically fail for proteins with longer sequences, while the evolutionary methods depend on the availability of a large number of suitably diverse sequence homologs.

Neither of the template-free strategies mentioned, however, can yet reach the reliability of homology modeling when a suitable template is available. Suitable, in this case, implies a structure with a similar overall architecture or “fold,” i.e., containing the same number or length of secondary structure elements arranged in the same relative positions in space. Notably, similar folds can be adopted by proteins with essentially no matching residues, in which case, the fold detection process becomes a matter of matching evolutionary patterns and structural elements rather than individual residues. While the classical search method, BLAST, for detecting sequence relatives was revolutionary in its speed, it nevertheless relies on exact sequence matching [1]. Its powerful cousin, the PSI-BLAST search, incorporates the evolutionary history captured after an initial BLAST search so as to increase the sensitivity in subsequent searches and thereby detect more distantly related proteins [2]. Even greater sensitivity can be achieved by tracking the likelihood of insertions and deletions in specific positions in the evolutionary record, through methods using Hidden Markov Models (HMM) as representations of the target or template, or both [34, 37]. The HMM profiles generated by the method HMMER [19], for example, comprise a set of aligned sequences combined with a secondary structure prediction averaged over all sequences in the set. In the case of the HHpred prediction tool [38], an HMM profile generated for the query sequence is scanned against a database containing the HMM of every structure in the Protein Data Bank (PDB) [4].

## Predicting the structural fold of NaPi-IIa transporters

### Identifying the repeat units of NaPi-IIa using hydrophobicity profiles and HMMs

As mentioned above, at the turn of this century, NaPi-II transporters were believed to contain two sets of transmembrane helices, or structural repeats, separated by an extracellular loop. Each of these sets of helices contains a copy of a motif with the sequence QSSS. Based on the differences in accessibility of these motifs to either side of the membrane, the structural repeats were suggested to adopt an inverted orientation with respect to the membrane plane [21]. However, the boundaries of these repeats were not clear (Fig. 1a). To determine which residues comprise each of the structural repeats and to establish if these two segments shared a common fold, we analyzed the hydropathy plots of these regions, taking advantage of the fact that proteins that share similar folds also share qualitatively similar hydrophobicity profiles [9]. After dividing the full-length profile at the position of the long loop, we then aligned the two fragments, revealing a clear relationship between the first ~ 180 residues of each of the fragments. The C-terminal fragment, however, contained an extension with two strong peaks likely corresponding to two additional transmembrane segments (Fig. 1b). Based on this analysis, we concluded that NaPi-IIa contains two repeat units (RU1 and RU2) comprising approximately five transmembrane segments each and that together these repeats constitute the core fold of NaPi-IIa (Fig. 1c). Moreover, from analysis of an HMM profile representing all NaPi-IIa amino acid sequences, we observed two distinctive conserved segments corresponding to RU1 and RU2, each containing the conserved QSSS motif, in addition to a short segment on the C-terminal end of the profile [9]. Using HHalign to align the HMM profile segments for the two conserved regions allowed us to assign the boundaries of the repeats to residues 86–256 and 335–489. The C-terminal residues 504–564 were predicted to contain two transmembrane helices (TM11–12) that are not part of the core fold, but instead are likely to be located at the periphery of the protein structure (Fig. 1c).

### Template detection using hydrophobicity profiles and HMM methods

The more detailed topology illustrated in Fig. 1c helped to delineate key features of the NaPi-II fold, but was still no substitute for a three-dimensional model of the transporter. Unfortunately, for many years, no structural templates for NaPi-II transporters could be identified using conventional methods such as PSI-BLAST, while the length of the protein (~ 560 residues) precluded template-free methods of structure prediction. Moreover, the peripheral helices predicted in the NaPi-II sequence were expected to further complicate the detection of distant sequence relationships. To address these challenges, Fenollar-Ferrer et al. [9] questioned whether the sequence search methods might be overlooking a suitable template and adopted a more sensitive approach, namely, scanning the HMM profile of NaPi-IIa against the protein databank (PDB [4]) using HHpred [18, 38]. This search identified several possible templates, albeit all assigned very low scores (*E* values ~ 1). Each of the putative templates was examined in detail, but one stood out: the Na^+^-coupled dicarboxylate transporter from *Vibrio cholerae*, VcINDY, which belongs to the dicarboxylate:sodium symporter (DASS) family. Not only did the VcINDY sequence align with the highest coverage (~ 62%) and identity (~ 7%) of all the putative templates, but the alignment also matched the conserved QSSS motif to a motif common to the DASS family. In the available structure of VcINDY [26], residues in this SNT motif contribute to the binding sites for Na^+^ and the anionic substrate, suggesting that, despite the low sequence identity between the two proteins, the binding regions are at least conserved. Moreover, the VcINDY structure contained a prominent inverted-topology structural repeat, as expected for NaPi-IIa.

The possibility that VcINDY could be a suitable template was put into question by the observation that its structure contains at least four more transmembrane segments than had been predicted for NaPi-II. Indeed, alignments of the full-length protein sequences using conventional methods suggested segment matching that was inconsistent with the known locations of the structural repeats and the core folds; specifically, those additional helices were inserted within the core of the NaPi-II transporter fold. This result reflects a common failure of alignment methods for very distant homologs of different lengths. Fenollar-Ferrer et al. [9] circumvented this issue by adopting a strategy similar to that used for identifying the repeats within the NaPi-IIa fold. Specifically, the repeats of each protein were separated out and aligned in a pairwise manner, with the aim of reducing the chances that core helices become aligned to peripheral helices. Both hydrophobicity profile alignments and HMM profile alignments of the RU1 and RU2 segments of NaPi-II and VcINDY suggested that the core fold of the two proteins is similar even though the first two transmembrane helices of each of the repeats of VcINDY have no counterpart in NaPi-II proteins (Fig. 1d, e). These four helices of VcINDY are in fact peripheral and not part of the core fold responsible for binding of Na^+^ or substrates [26].

Taken together, the high sequence coverage, the qualitatively similar hydrophobicity profiles, the reasonable correspondence between helices when the HMM profiles are aligned, and the matching of conserved residues important for the function of the protein corroborated the choice of VcINDY as a suitable template for homology modeling of NaPi-II transporters. This result also suggested a new, much more complex and detailed transmembrane topology (Fig. 1f).

### Building an initial model of human NaPi-IIa

**Fig. 2:**
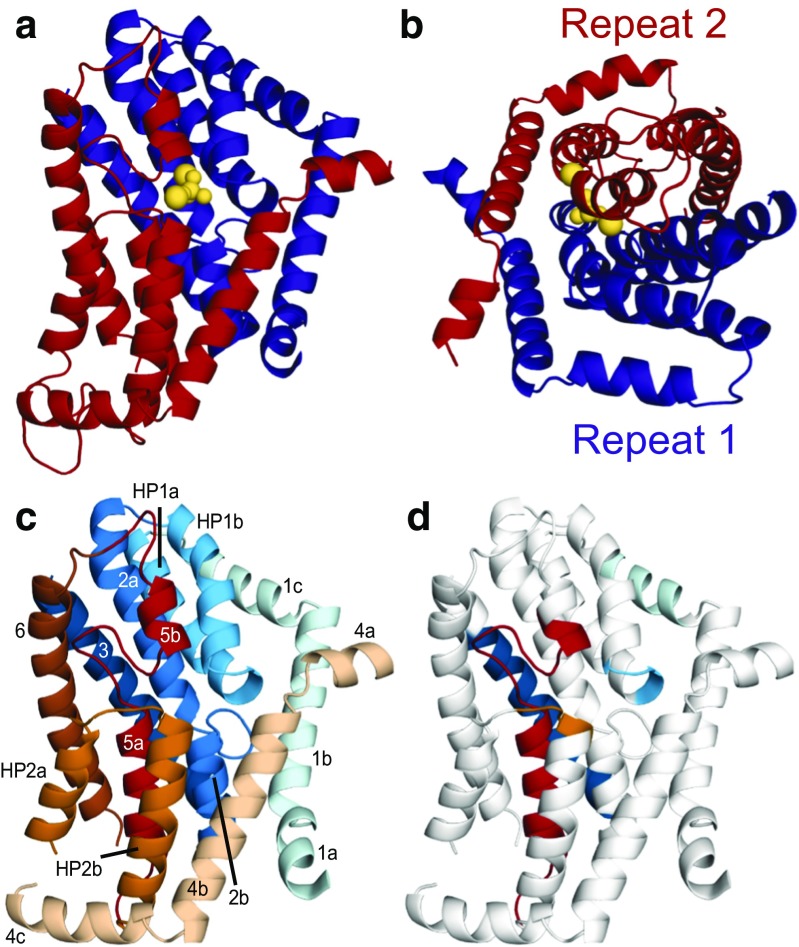
Structural fold of human NaPi-IIa, predicted by the homology model from Fenollar-Ferrer et al. in 2014 [9]. **a**, **b** The location of the pseudo-symmetric structural repeats 1 (blue) and 2 (red) in a structural model of human NaPi-IIa, viewed from **a** within the plane of the membrane with the cytoplasm towards the bottom or from **b** the extracellular side of the membrane. A phosphate ion and two sodium ions (yellow spheres) were modeled at the approximate axis of pseudo-symmetry, with positions based on those of substrates observed in the crystal structure of the template protein, VcINDY. **c** Same as **a**, but with helices colored individually. Helices in repeat 1 are colored shades of blue, according to Fig. 1d, while helices in repeat 2 are colored in shades of taupe through red, according to Fig. 1e. **d** Extent of the 2014 hNaPi-II homology model that could be validated by comparison to available biochemical accessibility measurements [7, 16, 20, 21, 23, 24, 32, 41, 42], with colored segments indicating the validated regions

A structural model of human NaPi-IIa (hNaPi-IIa) built based on the X-ray structure of VcINDY comprised transmembrane segments 1–6 from the predicted core transporter domain of hNaPi-IIa. That core consisted of an inverted-topology structural repeat, the two halves of which lay adjacent to each other, forming at their interface putative binding sites for the substrate and two sodium ions (Fig. 2a–c) [9]. The new topology obtained for hNaPi-IIa differed in notable ways from the earlier, simpler topologies. In particular, the new topology suggested the presence of two helical hairpins that do not fully span the membrane, as well as long non-helical elements that break up two membrane-spanning helices (Fig. 1f). The presence of these non-canonical elements provides an explanation for the unusual features in the hydrophobicity profile at these regions (residues 80–120 of each repeat). The architecture of this structural fold is such that these elements are exposed to aqueous solution (or to the rest of the protein), due to a framework in which the surrounding transmembrane helices shield them from the hydrophobic regions of the lipid bilayer.

The hNaPi-IIa structural model was also consistent with experimental data available in the literature at the time (Fig. 2d) [7, 16, 20, 21, 23, 24, 32, 41, 42]. In particular, cysteine-scanning mutagenesis (SCAM) data indicated high solvent accessibility of helix 1c, consistent with its location at the external surface of our model, and of loop L5ab, which is at the same depth as the substrate binding sites and, as a consequence, is accessible through the same aqueous pathway as the substrates [7, 23]. Similar experiments on Ser424 concluded that this residue was not exposed to the solvent [42], in agreement with a more buried position within HP2b in our model. Finally, the SCAM data obtained for TM3 [41] is in agreement with its lipid-lining, buried location in the hNaPi-IIa model.

### Predictions obtained from the initial model

The hNaPi-IIa model has modest resolution, as a consequence of the low sequence identity between hNaPi-IIa and VcINDY (Table 1). Nevertheless, the reasonable correspondence with the available experimental data suggests that the overall fold is correct. A notable consequence of this model is that the orientation of the helices in NaPi-II transporters within the membrane is the opposite of that of VcINDY. This difference in orientation arises from the facts that (a) the template contains an odd number of transmembrane segments at the N-terminal end before the repeats and (b) both proteins are oriented with their N-termini in the cytoplasm. As a consequence, although the known structure of VcINDY represents an inward-facing conformation in which the substrate binding site is close to the cytoplasm, the hNaPi-IIa model instead represents an outward-facing conformation [9].

**Table 1:** Available structural models of NaPi-II transporters

Protein	State	Modeled ligands	Sequence identity (%)	ProQM score*	Ref.
Human NaPi-IIa	Outward	P_i_, Na2, Na3	11	0.555	[9]
Human NaPi-IIa	Outward	P_i_, Na1, Na2, Na3	8	0.572	[8]
Flounder NaPi-IIb	Outward	P_i_, Na1, Na2, Na3	10	0.566	[30]
Flounder NaPi-IIb	Inward	P_i_, Na1, Na2, Na3	10	0.573	[30]

*ProQM scores range from 0 to 1, with 1 being the most similar to known membrane-protein structures [33]. For reference, the ProQM score of the structure of VcINDY used as a template (PDB code 4F35) was 0.675 when considering the entire structure and 0.643 after removing peripheral helices so as to match the elements present in the NaPi-II models. These models are reported in references [8, 9, 30]

Bolstered by the matching of the QSSS and SNT motifs, this model of hNaPi-IIa was also used to predict the binding sites for several of the substrates, including two of the three sodium ions required for transport. First, one of the sodium ions was modeled at the position of Na2 in VcINDY, where it could be readily coordinated by several suitable side chain and backbone groups from HP2ab and TM5 without additional modifications of the model. A second ion was tentatively modeled in the symmetric position, involving the equivalent segments from the other repeat, namely, HP1ab and TM2, consistent with the proposal from Wang and colleagues [26]. Again, a number of suitable side chain and backbone groups were available for cation coordination in this region without further adjustment to the model. Finally, inorganic phosphate was modeled in between these two cations, almost exactly at the symmetry axis [9], and similar to the location of the anionic substrate in VcINDY. In this position, the double negative charge on the substrate would be expected to be compensated by the sodium ions on either side.

These modeled binding sites are predictions based on primarily on homology, which helped to identify specific residues that might be responsible for binding. In addition, they raised the question of the location of a third sodium binding site, for which no equivalent was identified in the template.

## Refining the NaPi-IIa model by iterative modeling and experimental validation

### Refining models based on experimental data

The electrogenic isoform NaPi-IIa is characterized by a transport stoichiometry (Na^+^:HPO_4_^2−^) of 3:1 and by voltage-dependent transport kinetics [17]. It has been proposed that only two steps of the transport cycle are voltage dependent and that one of those two steps is the binding of the first Na^+^ ion to its binding site, which is referred to as Na1. The voltage dependency of transport by NaPi-IIa can be abolished by a single point mutation, D224G, rendering the transporter electroneutral [41]. Subsequent studies explored the role of this residue in NaPi-IIa as well as the equivalent residue in the electroneutral isoform NaPi-IIc in more depth, concluding that Asp224 potentially coordinates the Na^+^ ion in the Na1 binding site [3, 31].

**Fig. 3:**
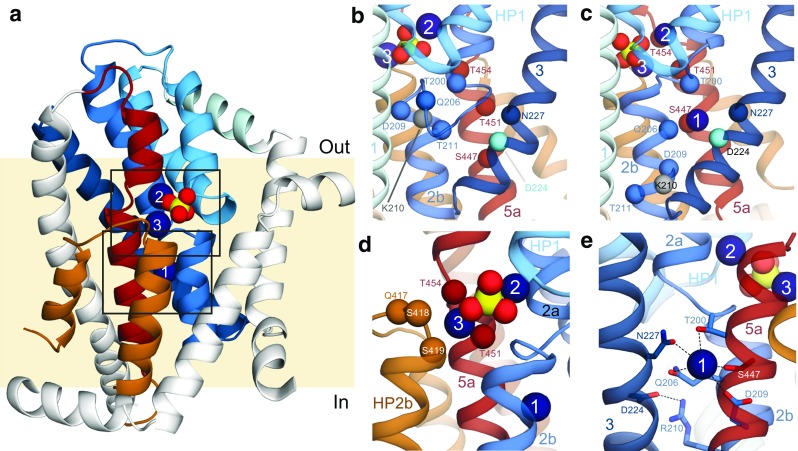
Predicted binding site residues in a refined structural model of human NaPi-IIa from Fenollar-Ferrer et al. in 2015 [8]. **a** Overview of a human NaPi-IIa model after refinement of several elements identified as contributing to the Na1 site. Helices with elements that have been validated by experimental data are colored according to Fig. 1d, e; white-colored segments remain to be validated. Bound phosphate (yellow and red) and sodium ions (deep blue) are shown as spheres. **b**, **c** The cytoplasmic half of the human NaPi-IIa model **b** before and **c** after refinement of the helices contributing to the proposed Na1 site. The threading of the residues in TM2a (sky blue) was adjusted so that residues with a Na1-site phenotype (Thr200, Gln206, Asp209, and Thr211, sky blue spheres) and Arg210 (gray sphere) were positioned closer to Asp224 (cyan sphere). At the same time, residues in TM5 (dark red) with a Na2-P_i_-Na3-site phenotype (Thr451 and Thr454, dark red spheres) were repositioned by adjusting the alignment of TM5 to its template. **d**, **e** Predicted substrate binding sites in human NaPi-IIa. **d** The Na2-P_i_-Na3 binding region is predicted to involve residues from HP1 (light blue), TM2 (sky blue), TM5 (dark red), and HP2 (brown). Putative coordinating residues in the Na3 site, Thr451, Thr454, Gln417, Ser418, and Ser419, are shown as spheres at the position of the Cα atom. **e** The Na1 binding site is predicted to involve residues from TM2b (sky blue), TM3 (dark blue), and TM5a (dark red). Residues found to be involved in binding (Thr200, Gln206, Asp209, Asn227, and Ser447) or to be in close proximity to the site (Asp224 and Arg210), according to mutagenesis and electrophysiological measurements, are shown as sticks

As mentioned, the model of hNaPi-IIa published in 2014 represented a state in which two Na^+^ ions were bound. Neither ion was within ~ 10 Å of Asp224, which was positioned at the cytoplasmic end of TM3, suggesting that neither of the two predicted cation sites corresponds to the Na1 site. Previous experiments had not been able to distinguish between the binding (or unbinding) events of the remaining substrates, namely, phosphate and the second and third cations. Consequently, the sites for the two bound ions, being adjacent to the phosphate in the model, had been assigned the labels Na2 and Na3. The location of the Na1 site, however, remained a mystery. To address this question, the 2014 hNaPi-IIa model was used to identify residues adjacent to Asp224 and TM3, with Na^+^-coordinating features, which might contribute to the Na1 binding site (Fig. 3b) [8]. A number of candidate residues were identified in the cytoplasmic halves of helices TM2b and TM5a. The human transporter was then subjected to mutagenesis at these positions, and the voltage-dependency of the steady-state and presteady-state kinetics was analyzed for each mutant [30]. Based on the similarity of their phenotypes to that of Asp224, it was concluded that residues Gln206, Asp209, and Ser447 also contribute to the Na1 binding site in hNaPi-IIa. In addition, residues Thr200 and Asn227 were found to play a similar role in the voltage-dependent steps of transport and therefore might be proximal in the protein structure, whereas Thr211 could be ruled out as a sodium-coordinating group. In addition, the experiments revealed that modifications at the position of Thr454 and Thr451 lead to a similar behavior as modifications of residues in the Na3 binding site.

Together, these data were used to refine the 2014 model in three main regions [30]. First, the alignment of TM2b was shifted so that residues Gln206 and Asp209 pointed towards TM3, while locating Thr211 further away and simultaneously positioning Thr200 to participate in either Na1 or Na2 binding sites (Fig. 3c). Next, the alignment of TM5 and TM6 was adjusted to position residue Ser447 closer to the known Na1-binding residues. As a consequence, residues Thr451 and Thr454 were placed in the Na3 binding site together with Gln417, Ser418, and Ser419 (from the QSSS motif of HP2; Fig. 3c, d). The resultant structural model is improved in the TM5-TM6 region (Table 1) according to the per-residue score from the empirical membrane protein model scoring function, ProQM [33]. The largest improvement in score was observed for TM6, probably due to the repositioning of three arginine side chains into the cytosol and away from the hydrophobic core of the membrane.

The final refined model, published in 2015, represents the hNaPi-IIa state in which the transporter is loaded with three sodium ions occupying the Na binding sites Na1, Na2, and Na3 and with a phosphate molecule interacting with sodium ions at the Na2 and Na3 sites [30]. Residues Thr200, Gln206, Asp209, and Asn227 coordinate one sodium ion at binding site Na1, while Arg210 and Asp224 form a salt bridge nearby. As mentioned above, the refinement also reorganized part of the Na3 binding site so that it is instead formed by residues Gln417, Ser418, Ser419, Thr451, and Thr454 (Figs. 3d, e).

For Na1, the final prediction involves residues from three different TM segments: TM2b, TM3, and TM5, which are far from one another in sequence. The fact that the experimental phenotype upon mutation of these residues is so consistent provides very strong support for the hypothesis that NaPi-II transporters share a common architecture with DASS family to which VcINDY belongs.

### Validation of the ion binding sites by structure comparison

Our computational studies of NaPi-II transporters indicate that this protein family has an overall architecture and core topology similar to that of VcINDY, even though the number of helices and their transmembrane orientation probably differ. More recently, X-ray structures of the transporters YdaH and MtrF [6, 39], which belong to the AbgT family, were compared with the structure of VcINDY, revealing a common two-domain fold—comprising the so-called transport and oligomerization domains—and demonstrating that the structures of the transport domains are particularly well conserved [40]. The structure of YdaH was of particular interest, as a Na^+^ ion was detected in the second structural repeat. The coordination of this ion involved residues from hairpin HP2 and the helix TM7. This position is symmetric to the site of the Na^+^ ion bound to repeat 1 of VcINDY and involves equivalent elements to the proposed Na3 binding site in the most recent model of NaPi-IIa, i.e., HP2 and TM5 [8]. Structural comparison of YdaH and NaPi-IIa by aligning their transport domains indicated that the predicted Na3 site in NaPi-IIa is in excellent agreement with the position of the Na3 site in YadH. Indeed, the ion at Na3 and the Cα carbons of residues Ser418 and Thr454 in NaPi-IIa are < 2 Å from the ion and equivalent groups in YadH. This observation provides strong validation of the refined hNaPi-IIa model [8].

### Examining conformational change using repeat-swap modeling

While the structural models of hNaPi-IIa reported in 2014 and 2015 provide important insights into the overall topology, they do not reveal a great deal about the mechanism by which the protein changes conformation so as to expose the binding sites to the opposite side of the membrane. For other secondary active transporters with inverted-topology repeats, it has been shown that a model of the opposite state than that observed experimentally can be constructed by exploiting the inherent asymmetry of the known structure [10, 12, 13]. Specifically, the asymmetry manifests as two distinct conformations for the repeat units. Thus, by exchanging their conformations (i.e., RU1 adopting the conformation of RU2, and vice versa), one can reveal the alternate state, i.e., with the binding site exposed to the other side of the membrane. In essence, this so-called repeat-swap modeling procedure is simply homology modeling, albeit using the two halves of the protein as templates for their counterparts simultaneously.

Repeat-swap modeling has been used to predict that VcINDY, the protein used as a template for modeling NaPi-IIa, uses a two-domain elevator-like mechanism [27]. In this dramatic conformational change, observed previously for another transporter containing hairpins, Glt_Ph_ [35, 36], the substrate binding site is moved in its entirety along with the rest of the transport domain, while another component of the transporter (typically the oligomerization interface) remains essentially static with respect to the membrane plane. The elevator-like conformational mechanism is quite distinct from mechanisms adopted by proteins such as LeuT, in which structural elements “rock” or make clam-shell-like movements around a central binding site. We note also that hybrid mechanisms, combining features of both rocking and elevator-like movements, may also be possible [43].

**Fig. 4:**
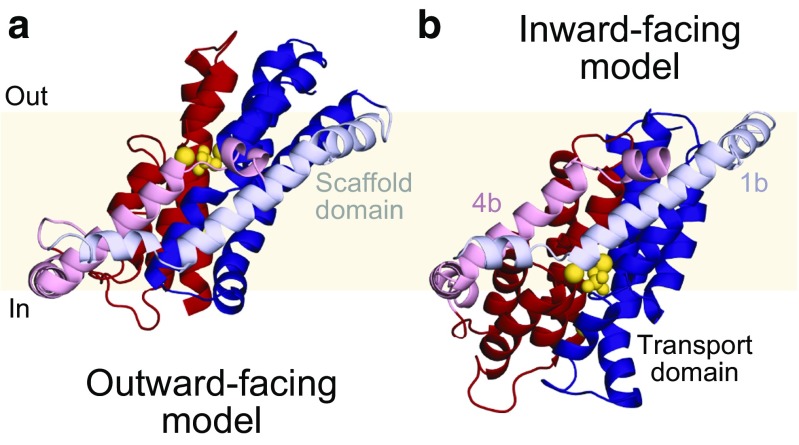
Prediction of the global elevator-like conformational change in NaPi-II transporters, based on the structural change expected for VcINDY [30]. Structural models of flounder NaPi-IIb are viewed from the plane of the membrane (yellow slab) and colored by repeat. Helices whose corresponding elements contribute to the fixed dimer interface in the template VcINDY are shown in lighter colors (scaffold, or oligomerization interface), whereas helices contributing to the more mobile transport domain and containing the substrate binding sites are shown in darker colors. Bound ligands are shown in yellow spheres. The outward-facing conformation (**a**) was built using a structure of VcINDY (PDB 4F35) as a template, whereas the inward-facing conformation (**b**) was built using a repeat-swapped model of VcINDY (one subunit in the dimeric model, PMDB code PM0080216) as a template

In view of their overall structural similarity to VcINDY, an inward-facing conformation of a NaPi-II transporter was predicted using the repeat-swapped model of VcINDY as a template (Table 1). Specifically, this inward-facing model was made for NaPi-IIb from flounder (fNaPi-IIb) [30]. In this model, the unwound element of TM5 and the loop connecting HP1a and HP1b are not exposed to the extracellular solution, unlike the outward-facing model of hNaPi-IIa, but instead are packing against helices TM1b and TM4b. Conversely, their symmetry counterparts, i.e., the unwound element of TM2, and the loop connecting HP2a and HP2b are exposed to the cytoplasmic solution, instead of packing against TM1b and TM4b. Illustrating the conformational change required the construction of an outward-facing model of fNaPi-IIb (as opposed to the available model of the human protein), which was built using conventional homology modeling using the crystal structure of VcINDY as a template [8]. Note that it would also have been possible to use an outward-facing model of NaPi-II as a starting point for repeat-swap modeling; the predicted conformational change would have been essentially the same. As expected, comparison of the models of the outward- and inward-facing conformations indicated that fNaPi-IIb also uses an elevator-like mechanism (Fig. 4). Based on the comparison with VcINDY, we propose that during this conformational change, helices TM1b and TM4b contribute to the presumed oligomerization interface, while the hairpins and unwound segments are included within the mobile transport domain.

Unfortunately, although the inward-facing NaPi-IIb model was of reasonable quality according to the ProQM score (Table 1), this model was limited as it is missing the extracellular loop connecting TM3 and TM4a, as well as the last two transmembrane helices, in addition to being a monomer (as the dimer interface is unknown). The absence of the long extracellular loop in particular prevented a conclusive comparison or validation based on voltage-clamp fluorometry measurements carried out to examine the conformational change [30]. Thus, although the biophysical measurements led to the conclusion that this protein undergoes a large movement similar to that predicted in an elevator-like mechanism, the details of the conformational change remain to be firmly established for the NaPi-II transporters.

## The future of NaPi-II structure-function studies

The structural models available for NaPi-II transporters have guided a number of experiments that have elucidated central features of their function, including residues contributing to substrate binding and an elevator-like conformational mechanism. Nevertheless, much remains to be learned, including a more detailed atomistic description of the key binding regions as required for drug discovery, as well as conformations of the protein in apo and partially occupied states, to help delineate the steps in the transport cycle. At present, all available models of NaPi-II transporters are limited to the core transmembrane elements and lack the C-terminal peripheral helices, the terminal elements, and the long extracellular loop that hosts the glycosylation sites. Moreover, in the absence of the peripheral helices, it is unclear exactly how the transporter would dimerize, although evidence from other elevator-like transporters indicates that the dimer interface would likely not involve elements of the transport domain. Additional structural data, even in the form of low-resolution cryo-EM maps, would be of great value in this regard, for example, by aiding with positioning of probes to examine transport dynamics and kinetics. Finally, resolving the terminal domain structures would provide key information relating to regulatory interactions with cytoplasmic proteins.

In the meantime, further modeling studies have the potential to provide important insights. For example, recently developed methods that leverage evolutionary-coupling information (see [28] for review) could provide restraints to complete the model of the protein, including contacts between the peripheral helices and those in the core, or even to refine helix-helix contacts within the core of the protein. As additional structures become available, e.g., of VcINDY in different conformations, or of more closely related proteins, these structures may be used as templates to build additional models that can guide experiments in unforeseeable, but exciting new directions. Whatever may be the case, these studies make clear that structure prediction can, and will continue to, offer powerful contributions when integrated closely with functional studies (see Forster IC et al. in this issue).

## References

[1] Altschul SF, Gish W, Miller W, Myers EW, Lipman DJ (1990). Basic local alignment search tool. J Mol Biol.

[2] Altschul SF, Madden TL, Schäffer AA, Zhang J, Zhang Z, Miller W, Lipman DJ (1997). Gapped BLAST and PSI-BLAST: a new generation of protein database search programs. Nucleic Acids Res.

[3] Bacconi A, Virkki LV, Biber J, Murer H, Forster IC (2005). Renouncing electroneutrality is not free of charge: switching on electrogenicity in a Na+−coupled phosphate cotransporter. Proc Natl Acad Sci U S A.

[4] Berman H, Henrick K, Nakamura H (2003). Announcing the worldwide Protein Data Bank. Nature Struct Biol.

[5] Biber J, Hernando N, Forster I (2013). Phosphate transporters and their function. Annu Rev Physiol.

[6] Bolla JR, Su CC, Delmar JA, Radhakrishnan A, Kumar N, Chou TH, Long F, Rajashankar KR, Yu EW (2015). Crystal structure of the Alcanivorax borkumensis YdaH transporter reveals an unusual topology. Nat Commun.

[7] Ehnes C, Forster IC, Bacconi A, Kohler K, Biber J, Murer H (2004). Structure-function relations of the first and fourth extracellular linkers of the type IIa Na+/Pi cotransporter: II. Substrate interaction and voltage dependency of two functionally important sites. J Gen Physiol.

[8] Fenollar-Ferrer C, Forster IC, Patti M, Knoepfel T, Werner A, Forrest LR (2015). Identification of the first sodium binding site of the phosphate cotransporter NaPi-IIa (SLC34A1). Biophys J.

[9] Fenollar-Ferrer C, Patti M, Knopfel T, Werner A, Forster IC, Forrest LR (2014). Structural fold and binding sites of the human Na(+)-phosphate cotransporter NaPi-II. Biophys J.

[10] Forrest LR (2013). Structural biology. (Pseudo-)symmetrical transport. Science.

[11] Forrest LR, Tang CL, Honig B (2006). On the accuracy of homology modeling and sequence alignment methods applied to membrane proteins. Biophys J.

[12] Forrest LR, Zhang Y-W, Jacobs MT, Gesmonde J, Xie L, Honig B, Rudnick G (2008). A mechanism for alternating access in neurotransmitter transporters. Proc Natl Acad Sci U S A.

[13] Forrest LR, Zhang YW, Jacobs MT, Gesmonde J, Xie L, Honig BH, Rudnick G (2008). Mechanism for alternating access in neurotransmitter transporters. Proc Natl Acad Sci U S A.

[14] Forster IC, Hernando N, Biber J, Murer H (2012). Phosphate transport kinetics and structure-function relationships of SLC34 and SLC20 proteins. Curr Top Membr.

[15] Forster IC, Hernando N, Biber J, Murer H (2013). Phosphate transporters of the SLC20 and SLC34 families. Mol Asp Med.

[16] Ghezzi C, Meinild AK, Murer H, Forster IC (2011). Voltage- and substrate-dependent interactions between sites in putative re-entrant domains of a Na(+)-coupled phosphate cotransporter. Pflugers Arch.

[17] Ghezzi C, Murer H, Forster IC (2009). Substrate interactions of the electroneutral Na+-coupled inorganic phosphate cotransporter (NaPi-IIc). J Physiol.

[18] Hildebrand A, Remmert M, Biegert A, Soding J (2009). Fast and accurate automatic structure prediction with HHpred. Proteins.

[19] Johnson LS, Eddy SR, Portugaly E (2010). Hidden Markov model speed heuristic and iterative HMM search procedure. BMC Bioinformatics.

[20] Karlin A, Akabas MH (1998). Substituted-cysteine accessibility method. Methods Enzymol.

[21] Kohler K, Forster IC, Stange G, Biber J, Murer H (2002). Identification of functionally important sites in the first intracellular loop of the NaPi-IIa cotransporter. Am J Physiol Renal Physiol.

[22] Kryshtafovych A, Monastyrskyy B, Fidelis K, Moult J, Schwede T, Tramontano A (2018). Evaluation of the template-based modeling in CASP12. Proteins.

[23] Lambert G, Forster IC, Stange G, Kohler K, Biber J, Murer H (2001). Cysteine mutagenesis reveals novel structure-function features within the predicted third extracellular loop of the type IIa Na(+)/P (i) cotransporter. J Gen Physiol.

[24] Lambert G, Traebert M, Hernando N, Biber J, Murer H (1999). Studies on the topology of the renal type II NaPi-cotransporter. Pflugers Arch.

[25] Lederer D, Shears D, Benoit V, Verellen-Dumoulin C, Maystadt I (2014). A three generation X-linked family with Kabuki syndrome phenotype and a frameshift mutation in KDM6A. Am J Med Genet A.

[26] Mancusso R, Gregorio GG, Liu Q, Wang DN (2012). Structure and mechanism of a bacterial sodium-dependent dicarboxylate transporter. Nature.

[27] Mulligan C, Fenollar-Ferrer C, Fitzgerald GA, Vergara-Jaque A, Kaufmann D, Li Y, Forrest LR, Mindell JA (2016). The bacterial dicarboxylate transporter VcINDY uses a two-domain elevator-type mechanism. Nat Struct Mol Biol.

[28] Nicoludis JM, Gaudet R (2018). Applications of sequence coevolution in membrane protein biochemistry. Biochim Biophys Acta.

[29] Olivella M, Gonzalez A, Pardo L, Deupi X (2013). Relation between sequence and structure in membrane proteins. Bioinformatics.

[30] Patti M, Fenollar-Ferrer C, Werner A, Forrest LR, Forster IC (2016). Cation interactions and membrane potential induce conformational changes in NaPi-IIb. Biophys J.

[31] Patti M, Ghezzi C, Forster IC (2013). Conferring electrogenicity to the electroneutral phosphate cotransporter NaPi-IIc (SLC34A3) reveals an internal cation release step. Pflugers Arch.

[32] Radanovic T, Gisler SM, Biber J, Murer H (2006). Topology of the type IIa Na+/P (i) cotransporter. J Membr Biol.

[33] Ray A, Lindahl E, Wallner B (2010). Model quality assessment for membrane proteins. Bioinformatics.

[34] Remmert M, Biegert A, Hauser A, Soding J (2011). HHblits: lightning-fast iterative protein sequence searching by HMM-HMM alignment. Nat Methods.

[35] Reyes N, Ginter C, Boudker O (2009). Transport mechanism of a bacterial homologue of glutamate transporters. Nature.

[36] Silverstein N, Crisman TJ, Forrest LR, Kanner BI (2013). Cysteine scanning mutagenesis of transmembrane helix 3 of a brain glutamate transporter reveals two conformationally sensitive positions. J Biol Chem.

[37] Söding J (2005). Protein homology detection by HMM-HMM comparison. Bioinformatics.

[38] Soding J, Biegert A, Lupas AN (2005). The HHpred interactive server for protein homology detection and structure prediction. Nucleic Acids Res.

[39] Su CC, Bolla JR, Kumar N, Radhakrishnan A, Long F, Delmar JA, Chou TH, Rajashankar KR, Shafer WM, Yu EW (2015). Structure and function of Neisseria gonorrhoeae MtrF illuminates a class of antimetabolite efflux pumps. Cell Rep.

[40] Vergara-Jaque A, Fenollar-Ferrer C, Mulligan C, Mindell JA, Forrest LR (2015). Family resemblances: a common fold for some dimeric ion-coupled secondary transporters. J Gen Physiol.

[41] Virkki LV, Forster IC, Bacconi A, Biber J, Murer H (2005). Functionally important residues in the predicted 3(rd) transmembrane domain of the type IIa sodium-phosphate co-transporter (NaPi-IIa). J Membr Biol.

[42] Virkki LV, Murer H, Forster IC (2006). Mapping conformational changes of a type IIb Na+/Pi cotransporter by voltage clamp fluorometry. J Biol Chem.

[43] Yu X, Yang G, Yan C, Baylon JL, Jiang J, Fan H, Lu G, Hasegawa K, Okumura H, Wang T, Tajkhorshid E, Li S, Yan N (2017). Dimeric structure of the uracil:proton symporter UraA provides mechanistic insights into the SLC4/23/26 transporters. Cell Res.

